# Evaluating Temporal Factors in Combined Interventions of Workforce Shift and School Closure for Mitigating the Spread of Influenza

**DOI:** 10.1371/journal.pone.0032203

**Published:** 2012-03-05

**Authors:** Tianyou Zhang, Xiuju Fu, Stefan Ma, Gaoxi Xiao, Limsoon Wong, Chee Keong Kwoh, Michael Lees, Gary Kee Khoon Lee, Terence Hung

**Affiliations:** 1 Institute of High Performance Computing, A*STAR, Singapore; 2 Nanyang Technological University, Singapore; 3 National University of Singapore, Singapore; 4 Ministry of Health, Singapore; Northeastern University, United States of America

## Abstract

**Background:**

It is believed that combined interventions may be more effective than individual interventions in mitigating epidemic. However there is a lack of quantitative studies on performance of the combination of individual interventions under different temporal settings.

**Methodology/Principal Findings:**

To better understand the problem, we develop an individual-based simulation model running on top of contact networks based on real-life contact data in Singapore. We model and evaluate the spread of influenza epidemic with intervention strategies of workforce shift and its combination with school closure, and examine the impacts of temporal factors, namely the trigger threshold and the duration of an intervention. By comparing simulation results for intervention scenarios with different temporal factors, we find that combined interventions do not always outperform individual interventions and are more effective only when the duration is longer than 6 weeks or school closure is triggered at the 5% threshold; combined interventions may be more effective if school closure starts first when the duration is less than 4 weeks or workforce shift starts first when the duration is longer than 4 weeks.

**Conclusions/Significance:**

We therefore conclude that identifying the appropriate timing configuration is crucial for achieving optimal or near optimal performance in mitigating the spread of influenza epidemic. The results of this study are useful to policy makers in deliberating and planning individual and combined interventions.

## Introduction

Past influenza pandemics and the recent H1N1 pandemic alert people the unpredictability and potentially overwhelming impacts of influenza outbreaks. While it is certain that the next pandemic will arrive in human societies, it is almost impossible to predict the virus type, transmission manner, and attack and mortality rates etc. Such unpredictability seriously challenges the public health system. Supplies of vaccine and pharmaceuticals may not be available or may be in shortage for a few months or even longer while a substantial number of infected cases has been reported. Under such critical circumstances, non-pharmaceutical interventions are usually considered in the first place, aiming at mitigating the spread and lowering the attack rate and fatality.

Workplaces and schools are both crucial community structures in epidemic control and mitigation planning. High contact rate and long contact duration in workplaces and schools may promote the transmission among workforce and school population. However, closure of workplaces causes significant disruption to economic activities and social functioning. Therefore a large-scale of workplace closure has seldom been implemented in the history of infectious disease control. In order to reduce contacts in workplace during epidemic, policy makers may seek alternative interventions, such as workforce shift. In workforce shift intervention, a portion (work team) of workforce is scheduled away from workplaces for a certain time span and then return by shifting with others. Workforce shift has been planned in real-life epidemic control. UK influenza contingency plan suggests 25% of employees taking 5–8 days off to enhance social distancing [1]; in the Singapore guideline of infectious disease control for workplace, dividing employees into work teams with minimum contacts between teams by shift system is suggested [2]. To our best knowledge, there are no studies on evaluating policies similar to workforce shift for influenza mitigation. We therefore investigate how effective team-based rotational workforce shift is.

Compared to workplace closure, school closure had been practiced more frequently and also widely evaluated in epidemic and pandemic control [3]–[6]. In a recent article [7], multiple aspects of school closure were reviewed, and it concluded that there are still many uncertainties on mitigation benefits of school closure as a public health policy. Historical school closure implementation data in real-world epidemic mitigation also showed contradictory conclusions, e.g., the encouraging results achieved in Israel [8] and the less encouraging ones in Hong Kong [9].

As workforce shift and school closure target different portions of the population, the combination of the two strategies may achieve better mitigation for influenza epidemic. On the other hand, however, as mass social distancing strategies, they may cause considerable economic and social costs. Any decision on intervention combination should be cautiously deliberated. This calls for quantitative evaluations on the effectiveness of combined intervention strategies.

Combined interventions for influenza epidemic have been evaluated widely in the literature. Germann et al [10], Carret et al [11] and Milne et al [3] assumed that combined interventions are implemented before the outbreak of epidemic and lasted until the end. Halder et al. [4] evaluated combined interventions with limited durations. Longni et al [12] and Ferguson et al [5] studied how different effectiveness levels and coverage of interventions could impact the attack rate and peak incidence. Halloran et al [13] and Rizzo et al [14] simulated the epidemic by implementing multiple strategies simultaneously at different time points with their own fixed durations. Duerr et al [15] tested the combination of two interventions in which one starts at the beginning of epidemic and the other may start at different time but always last until the end of the epidemic.

In this study, we evaluate a series of scenarios under workforce shift and its combination with school closure, with different trigger thresholds and durations. To our best knowledge, this is the first study evaluating combination effects of workforce shift and school closure for influenza mitigation. In comparison with the timing configuration in other studies, our study is different: 1) trigger thresholds of individual interventions can be configured in the combination independently; and 2) the duration of the combined interventions can be varied. Through simulation evaluations, we aim to provide a more comprehensive view on the impacts of temporal factors on social distancing interventions for influenza epidemic, helping to answer three key questions: a) *do combined interventions always outperform single interventions?* b) *how do trigger threshold and duration affect the effectiveness of combined interventions?* c) *does the implementation sequence in a combined intervention make a difference in its effectiveness?*

## Methods

Considering the importance of social structure in infectious disease spread, network-based models [1], [16], [17] have been commonly used for exploring the effectiveness of interventions in a heterogeneous-structured population for assisting policy makers to make proper decisions. In this work, we use a contact-network-based simulation model to carry out the evaluations based on Singapore's social structure. Specifically, we adopt an agent-based simulation model running on top of a social contact network. The network represents the statistical properties of interpersonal contacts which may lead to disease transmission in the specific community structure in Singapore. We evaluate workforce shift and its combination with school closure respectively, via extensive simulations with different trigger thresholds and implementation durations.

### Contact Network Construction

To address infectious disease spread with the consideration of the heterogeneity in social interactions, the most expressive approach is to form a structure of “network” by taking all individuals as vertices (or nodes) and their social connections as edges. We can further specify that an individual's social connections are the set of people with whom the individual may contact during the period when he or she is infectious. Thus, the disease transmission among the population can be simulated as the probabilistic propagation of viruses via the connecting edges in the contact network.

Generating a contact network representing for all individuals' contacts is complicated. To simplify the problem, we adopt a divide-and-conquer approach based on community structures of a typical society since social contacts most extensively take place in such community structures. For example, students contact with their peers at the schools; working adults contact with their colleagues at the workplaces; patients contact with healthcare workers and other patients at the hospitals, etc. We firstly determine the six types of community structures that are commonly reviewed in the literature [3], [17], [18] - *households*, *hospitals*, *schools*, *workplaces*, *shopping places* and *public transport.* Then we generate the community structures according to the statistics of them.

**Figure 1:**
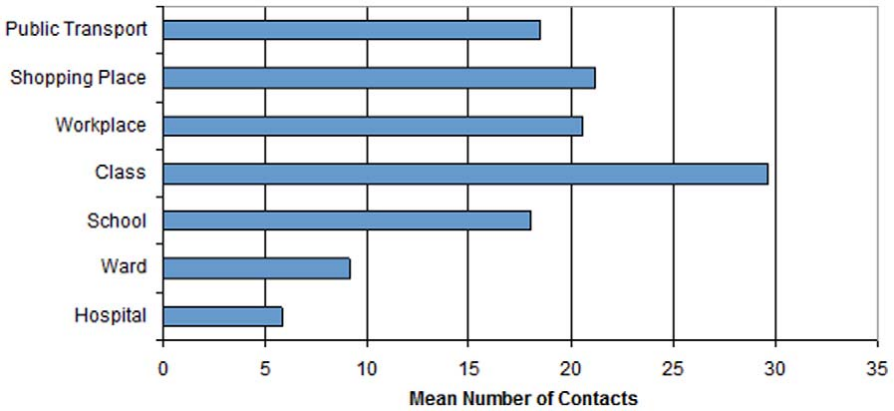
Mean numbers of contacts at different types of community structures (*class contacts refer to the contacts within the same class; school contacts refer to the contacts with the same school but different classes.* * A ward is a residential section in a hospital for serving hospitalized patients; ward contacts refer to the contacts between the patients in the same wards; and hospital contacts refer to the contacts between the patients in the same hospital but different wards*).

To lower the computational cost, the contact network is only comprised of 10% of Singapore population: age structure, household size distribution, characteristics of the modeled community structures have been retained proportionally in the simulated population to keep the epidemic trend consistent with that in the whole population. The sizes of communities in the network are obtained proportionally to the statistical numbers in the whole Singapore society. Specifically, a list of households is firstly generated based on the household size distribution. Subsequently, 35 schools are created proportionally according to the total number of students and school size distribution. Then each school is sub-divided into classes based on class size distribution. After that, students are assigned to schools and classes following the “enrollment in the nearby schools” policy, i.e., the students living nearby have a higher chance to be enrolled into the same school or class. Students in the same class may have more contacts (class contacts) and than those between students from the same school but different classes (school contacts), as shown in Figure 1. Similarly, 3 hospitals are constructed with sub-divisions – “wards” (i.e., sections in a hospital for accommodating hospitalized patients) based on hospital and ward size distribution (in term of number of beds) and bed occupation rate. Furthermore, ∼5,300 workplaces (equivalent to companies) are constructed based on the number of working adults, number of companies, and company size distribution, with no further sub-divisions; 10 shopping malls are created according to the survey data including the population size going for shopping, shopping frequency and daily traffic of malls, with no sub-divisions. Finally, a single structure of public transport is created as a single-layer giant component which includes all the commuters in the population.

Once the above community structures are constructed, individuals selected from the population pool are filled into each structure. The selection criteria are a set of rules defining the eligibility for a community structure. For example, age-based criteria can be used to define the enrollment to schools. Note that while an individual typically can be selected to join multiple different communities, some community enrollments are exclusive to each other. For example, an individual selected to be a patient staying in hospital should not participate in any of the school, workplace, shopping mall and public transport communities. However, his/her contacts within household may still remain as the visits from family members maintain such contacts. After assigning the subpopulation to a community structure, a local contact network is created by connecting the individuals of the subpopulation with the interpersonal contacts following a Poisson degree distribution [17] where the mean contact degrees are obtained from our social contact survey [18]. All the local contact networks are finally integrated into a global network.

The whole network generated in this study is comprised of ∼480000 vertices and ∼7.6 mil edges from 100,000 simulated households. Within the population, 11% are students, 61% are working adults, 0.2% stay in hospital, 22% visit shopping malls regularly and 34% use public transport on daily basis [19]. The social contact survey among the public of Singapore was conducted in 2008 with a survey form containing 45 questions. There are totally 1040 pieces of valid survey data collected. The extracted average numbers of contacts in different social locations are summarized in Figure 1 with the assumption that every household is fully connected [18].

Note that the contact network constructed in this study is unweighted. According to Newman [1], a disease will propagate equivalently in the population as a whole if all individual transmission probabilities are equal to the average transmission probability. By using the average transmission probability to replace each individual one, we simplify the transmission function incurred during the infection.

### Intervention Policies

Intervention polices are implemented to mitigate the transmission of disease. There are two categories of intervention: pharmaceutical and non-pharmaceutical interventions. Pharmaceutical interventions are mainly associated with vaccines and anti-viral drugs; and non-pharmaceutical interventions include isolation/quarantine, social distancing, etc. As vaccine production and anti-viral stockpiling often require substantial time after a pandemic occurs, non-pharmaceutical interventions are necessary to delay and dampen the pandemic before pharmaceuticals become available [20]. Workforce shift and school closure are the examples of social distancing interventions and will be evaluated in our work.

#### 1) Workforce Shift

In many countries, working adults occupy the largest portion of the population, and make close contacts with their co-workers in their daily activities. Closing workplaces has significant economic and social costs; so it is one of the least favorable choices that policy makers may consider. Another social distancing measure is workplace non-attendance, in which each worker has a 50% chance each day to choose either staying at home or attending to work. This policy is hard to implement as random and voluntary attendance of workers may cause chaos in the workplace.

Although workplace closure is seldom implemented in practice, policy makers do consider and suggest alternative workplace control, like workforce shift, for mitigating disease spread when necessary. In this study, we evaluate the workforce shift policy. Specifically, we assume that 1) each company or institution splits its employees into two work teams and implements 7-day rotation among the teams; 2) workforce shift is implemented immediately after the trigger threshold is reached; 3) for home-staying team members, all their contacts taking place in work places are removed from the contact network during the shifting period; and 4) workforce shift operation does not increase the contacts in other community structures.

#### 2) School Closure

School closure is a typical social distancing policy for mitigating the spread of infectious diseases among the student population. Generally, there are different types of school closure: 1) class closure, i.e., a class is closed if there are diagnosed cases; 2) individual school closure, i.e., a school is closed if there is a certain number of diagnosed cases, and 3) all-school closure, i.e., all schools are closed simultaneously if a threshold number of cases are diagnosed. All three types of school closure had been implemented in the real-world interventions in countries like Australia, UK, USA, and Japan to mitigate the spread of pandemic influenza [1], [16], [17].

In a previous study [18], all-school closure had been evaluated based on the same Singapore society setting with the consideration of different trigger thresholds and implementation durations. It was found that, in a cost-cautious situation where short intervention is preferred, school closure of 2-week should be implemented at a higher threshold (a later time); if reducing the epidemic size is the top priority, it is wise to implement a longer school closure (more than 6 weeks) as early as reasonable. In this paper, we evaluate combined workforce shift and school closure strategy.

### Models for Disease Spread and Intervention

**Figure 2:**
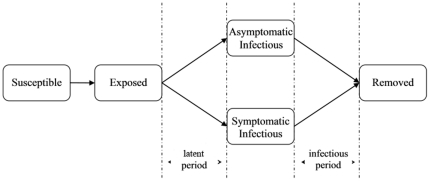
Dynamics of influenza progression within host individuals.

Figure 2 describes the host progression in the process of infectivity development of influenza illness within the host person. Any susceptible person has a chance (transmission probability) to be infected for every infectious contact s/he has. If the person (denoted as p) is infected, p is exposed but has no infectivity or any symptom yet. After the latent period, p becomes infectious (incubation period is assumed to be equal to latent period in the model). Specifically, p has a chance (symptomatic rate) to develop the clinical symptoms of influenza and turn into symptomatic infectious, or turn into asymptomatic infectious if without any symptoms. After the infectious period, p is finally removed, i.e. either recovered from influenza or dead.

Note that in our model, the probability of becoming infected goes up when a person is in contact with more infectious people, despite of the locations where the contacts occur. In lack of data about the infectivity regarding contact duration, we have assumed an unweighted contact network that propagates the disease with the average transmission probability along every edge of the network.

The focus of this study is on investigating the effectiveness of intervention polices under different scenarios. Specifically, we parameterize an intervention policy by six parameters: *trigger threshold, duration, target, control level, compliance rate and shift length*:

- *Trigger threshold* is a percentage of diagnosed (symptomatic) cases in the overall population, which is used to determine the starting time of intervention. For example, trigger = 0.1% means that an intervention will be implemented when 0.1% of the population is diagnosed as symptomatic cases of influenza.
- *Duration* refers to how long an intervention will be implemented.
- *Target* specifies what type of contacts is targeted by an intervention, such as school contacts, workplace contacts etc.
- *Control level* is used to differentiate the interventions performed at the different levels of a community structure, e.g. school-level closure and class-level closure.
- *Compliance rate* refers to the percentage of contacts that is removed by an intervention. As compliance rate is often affected by other interventions (e.g. workplace absenteeism may improve compliance rate during the school closure as adults will stay at home to take care of their children), we assume the 100% compliance rate for all-school closure to simplify our simulation scenarios.
- *Shift length* refers to the time span between team rotations.

## Results

The evaluation results of uncontrolled epidemic in the contact network serve as the baseline results. Different mitigation scenarios with different trigger thresholds and implementation durations are simulated based on the individual-based contact network simulation model. We then evaluate and compare the impacts of different temporal factors on the effectiveness of mitigation methods.

### Experiment Settings

The basic reproductive number, *R*_0_, is defined as the average number of secondary infections produced by a randomly selected infected person in a fully susceptible population [21]. Previous estimates of *R*_0_ in the past pandemic influenza were in the range of 1.5–2.3 [5], [22]–[25]. Unless otherwise specified, we assume *R*_0_ = 1.9 in our simulations, and adopt 66.7% symptomatic rate [26], 1-day latent period and 1.5-day mean infectious period [22], which are the same as those in a previous study [18]. By using Longini's approach [12], we approximate *R*_0_ = 1.9 empirically by tuning the base transmission probability. Specifically, we assume a scenario in which only a single individual is randomly infected where everyone else is susceptible yet not able to further transmit the disease, and count the number of secondary infections. The process is repeated for 10,000 times and *R*_0_ 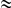
1.9 is then obtained as the average number of secondary infections. We found when the base transmission probability is 0.04, the empirical tests give the best approximation to *R*_0_ 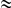
1.9 (95% Confident Interval (CI) 1.871–1.924), which yields the mean generation time of 2.5 days (95% CI, 2.489–2.508). The transmission probability is doubled to be 0.08 if the person is symptomatic infectious and meanwhile, half of his/her contacts are randomly removed due to self-isolation or self-shielding. Note that, in case of a new strain of influenza pandemic with unknown *R*_0_, the transmission probability in the network simulations can be tuned with assumed latent and infectious periods and symptomatic rate to get estimation of the new *R*_0_ by fitting to the reported epidemic curve.

In this study, we focus on examining the impacts of trigger threshold and duration length of interventions on the effectiveness of mitigating the influenza epidemic. The test scenarios are tabulated in Table 1. Each of those scenarios, including the baseline case, is simulated for 200 days and iterated for 100 times. All the results described in the following section are the average values of 100 simulation runs.

**Table 1:** Intervention scenario description.

Parameters	School Closure	Workforce Shift
*Trigger Threshold*	0.02%, 0.25%, 1.5%, 5%	0.02%, 0.25%, 1.5%, 5%
*Duration*	2,4,6,8,10 weeks	2,4,6,8,10 weeks
*Target*	school contacts	workplace contacts
*Control Level*	Schools	workplaces
*Compliance Rate*	100%	100%
*Shift length*	NA	7 days

Every simulation starts at *day* 0 with 10 infectious persons seeded into a susceptible population without prior immunity to the influenza virus. In our experiments, there are four trigger thresholds and five implementation durations available to choose for an intervention scenario. Hence there are totally 100 scenarios: 20 scenarios for workforce shift and 80 scenarios for the combined workforce shift and school closure (We assume that the individual interventions in each combination scenario share the same length of implementation duration.).

The effectiveness of interventions is examined by evaluating attack rate (AR), peak incidence (PI), and peak day (PD). Attack rate refers to the cumulative proportion of symptomatic cases of influenza infection in the overall population; peak incidence refers to the highest number of the daily incidence of symptomatic cases; peak day refers to the day when the peak incidence happens. In the public health perspective, attack rate indicates the size of epidemic and the overall burden on the public health system due to an epidemic; and peak incidence and peak day display the challenge to an effective response to patient surges in public health system.

### Influenza Spread without Intervention

**Figure 3:**
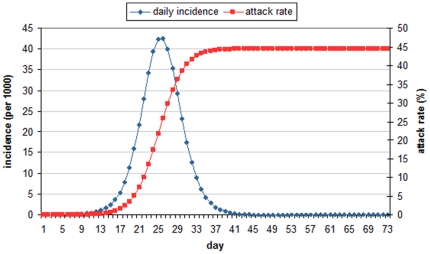
Average attack rate and daily incidence of baseline simulation in 100 runs (*R*_0_ = 1.9).

Figure 3 shows the average epidemic curves of 100 simulation runs for the case with no intervention. The epidemic reaches its peak at *day* 26 and fades out on *day* 73. The total attack rate *(AR)* is 44.47% (95% CI, 44.45%–44.48%); peak incidence (*PI*) is 42.45 per 1000 people (95% CI, 41.72–43.17). This result is comparable with 43.5% attack rate found in [10]. It is noted that the trigger thresholds {0.02%, 0.25%, 1.5%, 5%} are reached at *day* {7, 13, 17, 20} respectively.

### Impact of Workforce Shift

**Figure 4:**
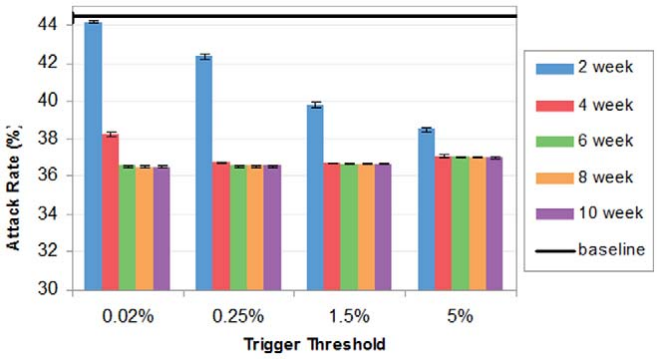
Attack rates with workforce shift.

As shown in Figure 4, the attack rates under workforce shift are in range from 36.51% to 44.21%, a 0.59% to 17.90% reduction compared to the baseline. The lowest attack rate takes place when the 10-week workforce shift is triggered at 0.02%. Consistent with the observation in school closure's results [18], the difference of attack rates at different thresholds but the same duration declines when the threshold increases. But the magnitude of the difference is larger for workforce shift compared to that for school closure. An extra 8.58% of the overall population can be saved from infections by choosing the appropriate trigger threshold for 2-week workforce shift, in comparison to 2.33% for 2-week school closure [18].

**Figure 5:**
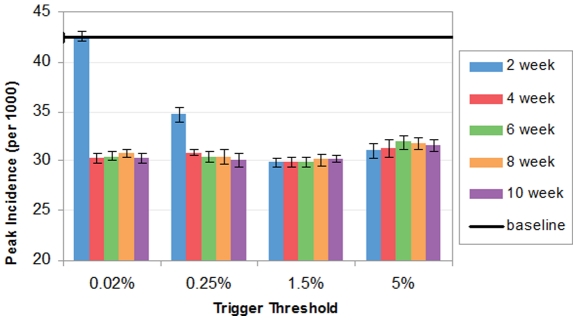
Peak incidence with workforce shift (per 1000 people).

Figure 5 shows that workforce shift has the remarkable impact of suppressing the peak incidence of influenza epidemic. The peak incidences under workforce shift range from 29.87 to 42.27 per 1000 people, a 0.04% to 29.63% reduction compared to the baseline. The lowest peak incidence occurs when the 2-week workforce shift is triggered at 1.5%. It is noted that 4 weeks are sufficiently long for reducing the peak incidence as no additional reduction is gained by extending the intervention.

**Figure 6:**
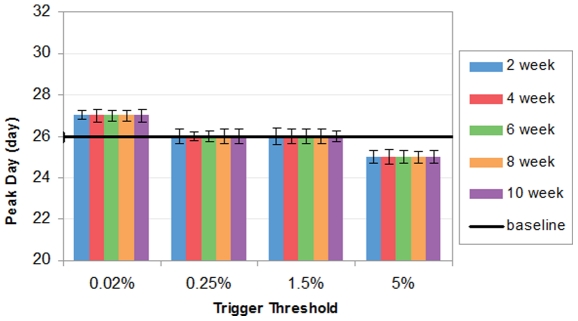
Peak attack day with workforce shift.

**Figure 7:**
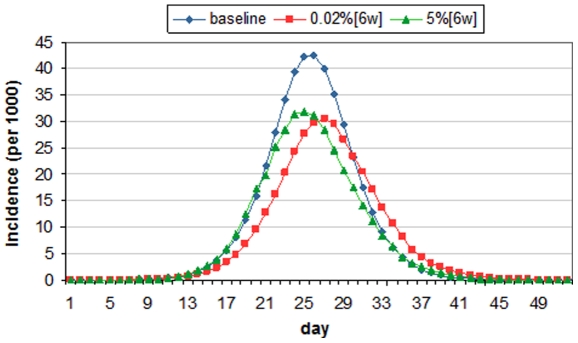
Daily symptomatic incidences from day 1 to 52, from baseline v.s. 6-week workforce shift triggered at 1.5% and 5% respectively.

Figure 6 shows that workforce shift has a mixed impact on peak day. Consistent with peak day results for school closure, varying duration makes no effect on peak day; and trigger threshold is the dominant factor deciding peak day. When trigger threshold rises from 0.02% to 5%, a consistent decline of peak days is observed. It could be explained that when workforce shift is implemented at a higher threshold, a larger number of the population has been infected and more potential transmissions will be blocked. Therefore, it sooner reaches the cutoff point at which the disease is unable to sustain the growth trend of incidences, so the peak would occur earlier. On the other hand, when workforce shift is implemented at a lower threshold, there are fewer infectious cases within the population and the amount of susceptible contacts left is still tolerable to maintain the chain of infections. Therefore, the daily incidence could be still growing but at a lower pace, consequently leading to a later peak day. Figure 7 shows divergent impact of workforce shift on peak day. 6-week workforce shift triggered at 5% advances the peak incidence by 1 day compared to the baseline; on the other hand, 6-week workforce shift triggered at 0.02% reaches the peak incidence 1 day later than the baseline.

### Impact of Combined Workforce Shift and School Closure

We then examine the combined intervention of workforce shift together with all-school closure. We are interested in the effectiveness of the combined intervention as well as the impact of the temporal sequence of individual interventions in a combination.

**Figure 8:**
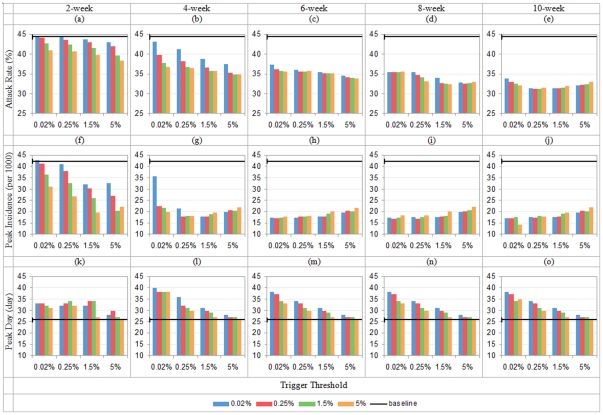
Total attack rate, peak daily incidence and peak attack day with hybrid control (*R*_0_ = 1.9; x-axis shows school closure's triggers, colored bar indicates workforce shift's triggers; in each row, duration = 2/4/6/8/10 weeks from left to right).

Figure 8 A–E show that the lowest attack rate (*AR*) under the combined intervention is 31.17%, achieved when workforce shift and school closure are both triggered at 0.25% and lasted for 10 weeks. In the single interventions, the lowest *AR* is 40.42% for all-school closure and 36.51% for workforce shift, both happen at 10-week duration and 0.02% trigger threshold. 8.01% of population can be further saved from the infection by applying the combined intervention compared to the single interventions.

Figure 8 F–J show that the lowest peak incidence (*PI*) occurs when 10-week workforce shift and school closure are triggered at 5% and 0.02% respectively. Compared with the lowest *PI* from single interventions (30.75 from school closure and 29.87 from workforce shift), the combined intervention is able to further reduce *PI* to 14.27.

Figure 8 K–O show that the combined intervention can delay the peak day (*PD*) by 14 days compared to the baseline. It is much longer than *PD* delay in individual interventions, i.e. 5-day delay by school closure and 2-day delay by workforce shift.

In the followings, we summarize our results in an attempt to answer the three questions asked in the earlier section:

#### (a) Do combined interventions always outperform single interventions?

It is commonly believed that combined interventions will outperform single interventions. But we notice some cases in which combined interventions lead to higher attack rates than single interventions at the same trigger threshold and duration. The worst case is observed when the 4-week workforce shift and school closure are both triggered at 0.02%. If we apply only workforce shift at 0.02% threshold with a 4-week duration (Scenario A – single intervention), the AR is 38.25%; on the other hand, AR from the combined intervention (Scenario B – Combined intervention) is 43.12%, which is 4.87% higher.

**Figure 9:**
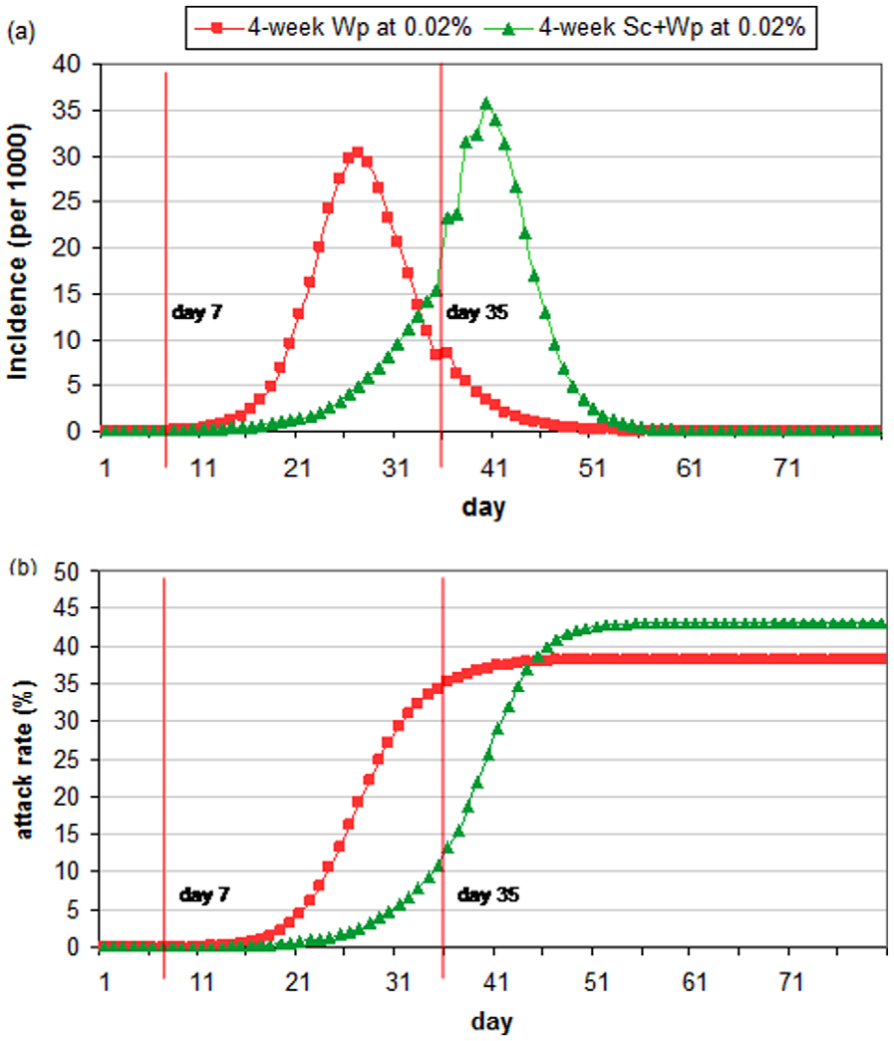
Comparison on daily incidence (A) and attack rate (B): red line denotes 6-week workforce shift (Wp) triggered at 0.02%; green line denotes 6-week school closure + workforce shift (Sc+Wp) triggered at 0.02%.

Figure 9 further describes what happens in *Scenarios A* and *B*. On *day* 7, the trigger threshold (*t* = 0.02%) is reached and the epidemic curve of the combined intervention grows much slower than the single intervention because more contacts have been removed and chance of infection is lower. On *day* 35, the interventions in both scenarios end and the removed contacts are restored. Because the growth of infected cases is much slower in *Scenario B*, there is more susceptible left in the population. Specifically, on *day* 35, 49.65% and 85.88% of population are susceptible in *Scenarios A* and *B* respectively. This nearly doubled size of susceptible population allows more disease-causing contacts and higher chance of infection in *Scenario B* compared to those in *Scenario A*, leading to the divergent developments of the epidemic after *day* 35 – the incidence continues to decline and gradually fades out in *Scenario A*; and oppositely in *Scenario B*, the incidence number grows exponentially until *day* 40 and a large number of infections take place after the intervention.

It is observed that 11 out of 16 combined scenarios of 2-week intervention underperform 2-week single interventions; 7 of 16 scenarios of 4-week interventions and 1 out of 16 scenarios in 6-week interventions lead to similar observation. Apparently combined interventions with a longer duration (> = 6 weeks) are less prone to underperform, meaning that combined interventions have to be maintained long enough to prevent the rapid spread of influenza after the intervention period.

#### b) How do trigger and duration affect the effectiveness of combined interventions?

The performance of combined interventions can be affected by both trigger and duration. When the duration increases, AR and PI decline consistently. When trigger threshold rises, AR and PI drop if the duration is shorter than d weeks (d = 8 for AR; d = 4 for PI); if the duration is longer than d weeks, AR and PI increase instead. In Figure 8 E and G, convex curves clearly show the existence of the above trends. For the peak incidence time, the PD drops when the triggers rises with d> = 4weeks. It also shows that a longer duration of intervention (>4 weeks) does not bring in any further delay of the peak incidence time.

#### c) Does the implementation sequence in a combined intervention make a difference in its effectiveness?

The temporal implementation sequence of individual interventions within the combined strategy may also affect the outcome of intervention. The maximal differences of the attack rates among sixteen threshold combinations are {6.13%, 8.24%, 3.47%, 3.21%, 2.59%} with {2, 4, 6, 8, 10}-week durations respectively. When duration is less than or equal to 6 weeks, the performance of the synchronized interventions (two individual interventions start from the same threshold) improves when the trigger rises. With longer control durations, the trend is not retained anymore. Comparing to the asynchronized combinations (individual interventions start at different thresholds) with the same duration, the relative performance of synchronized interventions turns from “underperformance” to “outperformance” when their triggers rise from 0.025% to 5% subject to the condition that the duration is within 8 weeks. When the duration is longer than 8 weeks, synchronized interventions underperform in most of the scenarios and hence it is wise to start them at different thresholds in the implementation.

For asynchronized combinations, the sequential order of implementing single interventions can affect the *AR* as well. We term two combined strategies with swapped trigger thresholds of the two individual interventions as a pair of symmetric strategies. The maximal differences in attack rates between a pair of symmetric strategies are {2.13%, 1.31%, 1.55%, 2.73%, 1.66%} for {2, 4, 6, 8, 10}-week durations respectively. It is observed that school closure should be implemented later when duration is less than 4 weeks; and workforce shift should start later when duration is longer than 4 weeks.

### Sensitivity Test on Values of R_0_

**Figure 10:**
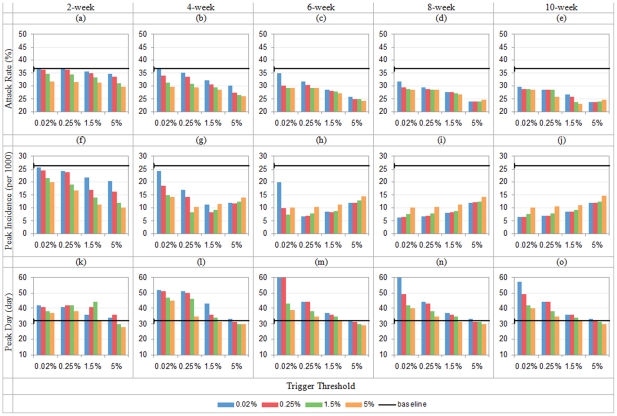
Total attack rate, peak daily incidence and peak attack day with hybrid control (*R*_0_ = 1.5; x-axis shows school closure's triggers, colored bar indicates workforce shift's triggers; in each row, duration = 2/4/6/8/10 weeks from left to right).

**Figure 11:**
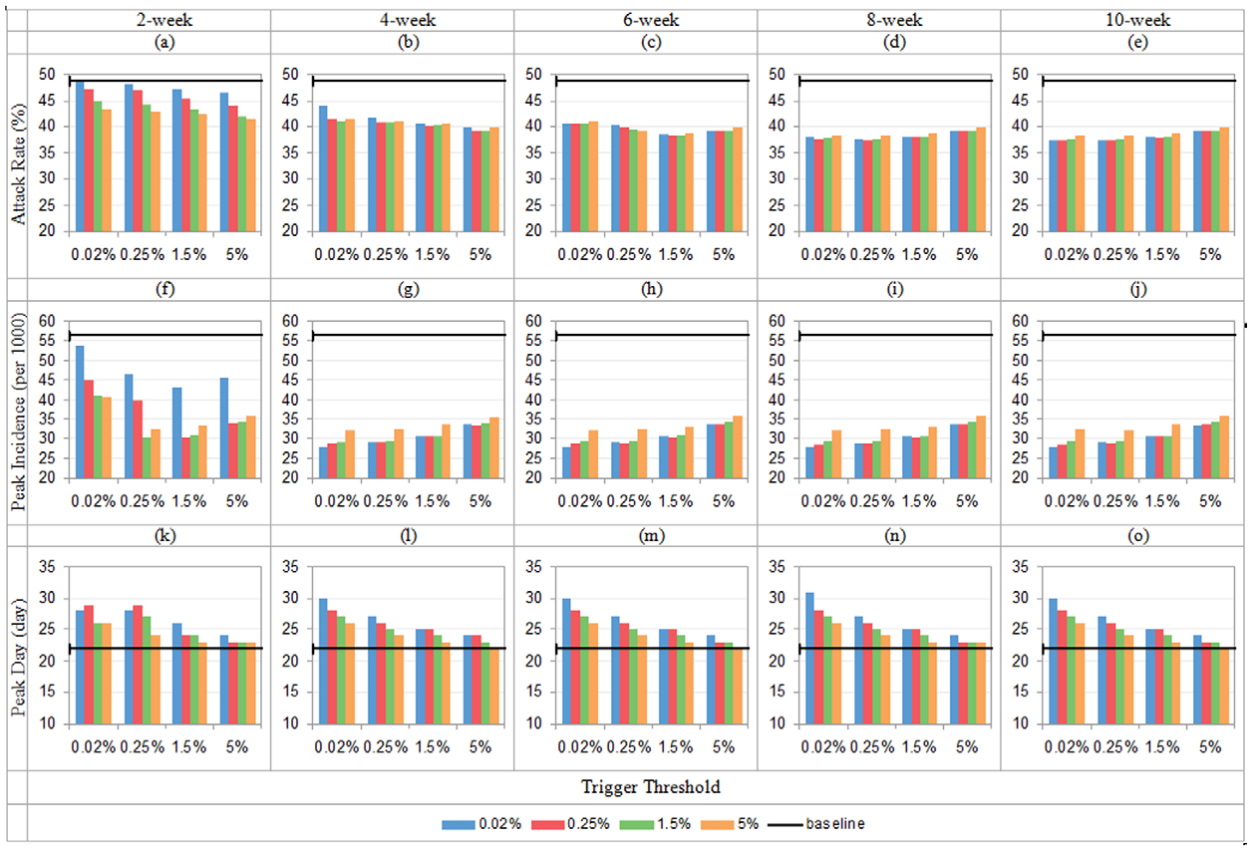
Total attack rate, peak daily incidence and peak attack day with hybrid control (*R*_0_ = 2.3; x-axis shows school closure's triggers, colored bar indicates workforce shift's triggers; in each row, duration = 2/4/6/8/10 weeks from left to right).

The results of temporal effects in the combined interventions of school closure and workforce shift are based on *R*_0_ = 1.9. To examine if our conclusions hold for other *R*_0_ values, we tested on different cases where *R*_0_ = 1.5 and 2.3. Similar to Figure 9, Figures 10 and 11 show the effectiveness of the combined interventions at different pairs of thresholds and durations for *R*_0_ = 1.5 and 2.3 respectively.

The results are consistent with our findings based on *R*_0_ = 1.9. Specifically, the worst combination happens when both school closure and workforce shift are implemented at 0.02% for 2 weeks. It yields 36.61% attack rate, 25.71 peak incidence (per 1000 people) on day 28 for *R*_0_ = 1.5; 48.52% attack rate, 53.88 peak incidence (per 1000 people) on day 28 for *R*_0_ = 2.3. The majority of single interventions, either school closure or workforce shift, show significant impact, except for 2-week school closure or workplace shift at 0.02% threshold.

In Figure 10 & 11, we also can observe the significant impact by adjusting temporal settings of the combined interventions. When *R*_0_ = 1.5, attach rate ranges from 36.90% down to 22.97% (37.8% reduction); peak incidence (per 1000 people) ranges from 27.20 to 6.12 (77.5% reduction); and peak day varies from 28 days to 76 days (171.4% increase). When *R*_0_ = 2.3, attach rate is in range from 48.67% down to 37.21% (23.5% reduction), peak incidence from 55.43 down to 27.73 (50.0% reduction), and peak day from 22 days to 31 days (40.9% increase). The observations suggest stronger impact of temporal factors for a lower value of *R*_0_.

For asynchronized combinations, the maximal differences in attack rates between a pair of symmetric strategies are {2.88%, 2.26%, 4.03%, 4.68%, 4.86%} for {2, 4, 6, 8, 10}-week durations where *R*_0_ = 1.5, and {3.49%, 1.69%, 2.14%, 0.85%, 0.9%} where *R*_0_ = 2.3. Again the observation is that when *R*_0_ is lower, switching the order in a combined intervention could make more significant difference. It is also interesting that the difference is particularly significant when duration is short (2 weeks) for all the three values of *R*_0_.

### Study on Weekend Effect

So far we have been adopting only the contact patterns during weekdays in our study. In urban life, however, social contact patterns may be significantly different during weekends. For example, the contacts in shopping malls may increase while contacts within workplace/schools may decrease. Such changes are terms as weekend effect in the context, which recurs for 2 days (Saturday and Sunday) in every week.

We conduct simulation to evaluate the impact of weekend effect. Specifically, we assume that school contacts are reduced by 50% and workplace contacts by 70% during the weekends compared to those during weekdays, and meanwhile shopping mall contacts are increased by 35.79% according to our survey data. Numerical experiments are then repeated at *R_0_* = 1.9 with the same configurations as listed in Table 1 for evaluating combined workforce shift and school closure. Our simulations are assumed to start on Monday; and when workforce shift intervention or school closure intervention is exercised, the population involved in the intervention will follow intervention arrangement regardless of weekday or weekend.

**Figure 12:**
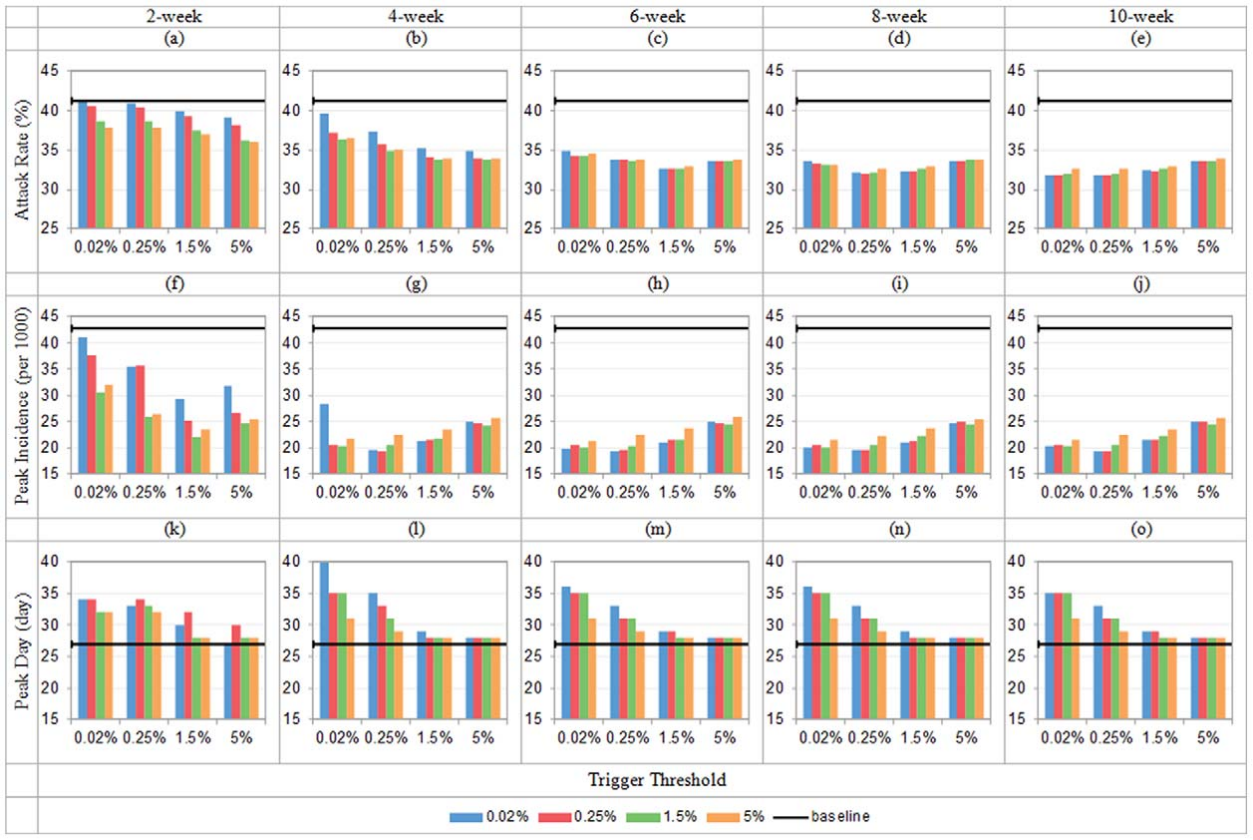
Total attack rate, peak daily incidence and peak attack day under hybrid control with the weekend effect (*R*_0_ = 1.9; x-axis shows school closure's triggers, colored bar indicates workforce shift's triggers; in each row, duration = 2/4/6/8/10 weeks from left to right).

Figure 12 shows the spread dynamic after introducing weekend effect. Compared to the experiments shown in Figure 8 without considering weekend effect, there exist similar patterns while varying thresholds and durations of the combined interventions. Meanwhile, however, we can observe the impact of weekend effect: the baseline attack rate under the weekend effect falls by 3.21% compared to the original one; peak incidence is only of a 0.18% difference; and peak day postpones by 1 day. Such results may be interpreted: the total removal of contacts from schools and workplaces is more than the contacts increased in shopping malls in the weekends.

When comparing the individual scenarios of the combined interventions, we find that the impact of weekend effect diminishes gradually with the increase in duration of interventions. This may be due to the enforcement on the control effect by the interventions from weekdays to weekends, i.e., weekend effect may be overridden by the control. For example, a part of weekend effect – 50% removal of school contacts in weekends may be overridden by school closure intervention and the 100% removal would happen during the whole period of school control. Therefore, the shorter the inventions are, the more notable the weekend effect is. The most notable decline in attack rate under the weekend effect is spotted in the 2-week intervention scenarios with the average reduction of 3.44% compared to the baseline of weekend effect.

## Discussion

Using an individual-based simulation model based on the social community structure of Singapore, we investigate the effectiveness of workforce shift and its combination with school closure as means to mitigate the spread of influenza. Specifically, the impacts of interventions have been investigated through evaluating the total attack rate and daily incidence as well as the delay of peak incidence time quantitatively.

Both workforce shift and school closure are social distancing measures that aim to reduce disease-causing contacts between individuals so as to reduce consequent secondary infections. As the production of vaccine and stockpiling of anti-viral drugs usually take considerable time, the shortage of pharmaceuticals has ever been the challenge in the preparedness planning for pandemic influenza and might not be ready at the time of influenza outbreak.

Our simulation results show that both workforce shift and school closure are able to lower attack rate and daily incidence as well as delaying the epidemic in most intervention scenarios. Such social distancing through enforcement from administration is necessary to mitigate the diffusion of influenza virus among the communities, especially when a large number of asymptomatic cases exist.

Our experiments provide guidance on choices of trigger threshold and length of duration for implementing school closure, workforce shift and their combination intervention measures. These results will be relevant to future contingency plan for influenza pandemic, which is estimated to be more pathogenic and might have higher case fatality rates than that shown in 2009 H1N1 pandemic flu [4]. We find that the durations of 8 weeks and 6 weeks are sufficiently long for workforce shift and school closure respectively. Short interventions should be implemented after a longer delay since outbreak; in contrast, long interventions should start as early as reasonable. The cutoff values between long and short duration are 6-week for school closure and 4-week for workforce shift, if lowering the attack rate is the priority.

Comparing the effect of workforce shift with school closure, we observe that workforce shift is generally more impactful. One of the main reasons is because of the difference in the number of people that can be affected by school and workplace interventions. In our contact network, school closure removes the school contacts from ∼53,000 people; workforce shift affects ∼148,000 people at any time during the intervention. So there is around 2.8 times more population controlled in the workforce shift.

Furthermore, we examine combined interventions as temporal combinations of single policies. We fix the duration shared by single policies in combination for simplicity; but allow different trigger thresholds so that the two policies may be implemented either one after another or at the same time. Our results show that the combined interventions do not always outperform the single interventions while varying trigger threshold and duration. It is shown that short closures (less than 6 weeks) are more prone to underperformance compared to that of the workforce shift only. Secondly, we observe that switching the order of single policies in combination can make a difference in the effect of intervention. Planning multiple interventions in the appropriate order is able to strengthen the mitigation to the spread of epidemic without significant additional cost.

Among all choices of combined interventions examined, the near-optimal policy happens when all workforce shift and school closure are both implemented at the 0.25% trigger threshold and lasted for 10 weeks (31.17% attack rate; peak incidence of 17.42 per 1,000 people at *day* 33).

Enforcing a social distancing policy always associates with considerable cost, on both economic and social aspects. For example, the major cost of school closure comes from absenteeism of working parents who have to stay home to take care of their children. A UK study [27] estimated 16% of UK workforce as the main carers of dependent children and likely to be absent due to school closure. This percentage could further climb to 30% if counting healthcare workers only, meaning more absenteeism could happen in public healthcare system which has been already stressful during an epidemic. Besides, there are also problems about social justice, ethical issues etc as the social consequence of school closure [7]. On the other hand, workplace distancing measures like workforce closure might lead to an abrupt shortage of manpower, lower productivity and inevitable economic loss. As an alternative to workplace closure and uncontrolled absenteeism, workforce shift might be an option for disease containments. Nowadays, accessible infrastructure for telecommunication is widely available at many workplaces and homes. Tele-working has become feasible and can be equipped in advance along with the planned workforce shift. It makes workforce shift with longer duration more acceptable. The planned workforce shift would help companies and other institutions to minimize the impact of mass absenteeism and sustain the usual business and production as much as possible.

In lack of information about the compliance rate of school closure, we have assumed a 100% compliance rate in all relevant intervention scenarios in this study. However, in a real-world school closure, the student compliance rate for social distancing may be at a lower value. The compliance rate of students for social distancing would be increased when implementing workforce shift together with school closure. We ignore the variation of compliance rate for not complicating the analysis on the combined interventions. A higher compliance rate is definitely preferred in real-world interventions and needs the coordination among education agency, health agency and communities to achieve.

Considering the network dynamics in weekends, we study the disease spread under the weekend effect. As schools and many workplaces are closed during the weekends, the contacts between schoolmates or between colleagues may be partially removed (Schoolmates or colleagues may hang out together during the off days) [28] but the shopping mall contacts may increase. In our experiments of the weekend dynamics, we find that the weekend effect does not bring significant variation to the baseline epidemic curve compared to that of the original setting without considering the weekend effect. It is worth noting however that due to the lack of real-world data, we have made assumptions on the reduction degrees of school/workplace contacts during weekends. In our future work, we will keep collecting real-world dynamic contact parameters of Singapore, and further evaluate the temporal effect of social distancing in dynamic settings.

The evaluation of intervention scenarios in this study is based on Singapore's social structure. The results presented here should be interpreted with the following caveats in mind. First, the Singapore community is not a closed system. There are millions of visitors arriving in Singapore (e.g., a peak of 10 million visitors in 2007). Singapore has a population size of around 4.9 million. The large volumes of visitors flowing into the country implicitly indicate that the influence of imported cases should be considered when planning intervention strategies. However, the influence of visitors is not considered in our research as we focus on investigating and comparing the effectiveness of the individual and combined intervention scenarios. We note that it is desirable to further analyze the influence of visitors on the disease spread in the community for combating future pandemic. Further, Singapore is a highly urbanized city and its population density is among the top in the world, which will definitely lead to high contact numbers in different community structures. The best intervention scenario in terms of control timing may vary when the social structure is drastically different from the one studied in this paper, as the heterogeneity of social structure is a significant factor affecting disease spread and consequently affecting mitigation planning strategies as well.

### Conclusion

Though the combined intervention strategy outperforms its individual strategies in most cases, it is found that combined intervention strategies underperform its individual intervention strategies under inappropriate timing configurations. Our results suggest that trigger threshold and duration are critical to the effectiveness of the combined intervention, specifically, for lowering attack rate and daily incidence as well as having a longer peak delay. Our studies also show that the implementation order of individual interventions in the combination could affect the effectiveness of combined interventions as well. Exploring correct timing configuration is therefore crucial to achieving optimal or near optimal effect of mitigation for influenza epidemic. Such an evaluation is recommended for assisting policy makers in influenza preparedness planning with their specific situation and constraints.
